# Anticoagulant Related Nephropathy Induced by Dabigatran

**DOI:** 10.1155/2018/7381505

**Published:** 2018-12-09

**Authors:** Nazia Sharfuddin, Mahra Nourbakhsh, Alan Box, Hallgrimur Benediktsson, Daniel A. Muruve

**Affiliations:** ^1^Department of Medicine, University of Calgary, Canada; ^2^Department of Pathology & Laboratory Medicine, University of Calgary, Canada; ^3^Snyder Institute for Chronic Diseases, University of Calgary, Canada

## Abstract

We describe a case of biopsy-proven dabigatran related nephropathy in a patient without underlying IgA nephropathy. To date, dabigatran related nephropathy was only reported in patients with concurrent or undiagnosed IgA nephropathy, suggesting that it may predispose patients to dabigatran associated injury. The patient is an 81-year-old woman with multiple medical comorbidities, including nonvalvular atrial fibrillation, who was anticoagulated with dabigatran. She presented to hospital with acute kidney injury in the setting of volume overload. Her estimated glomerular filtration rate decreased from a baseline of 57 mL/min/1.73 m^2^ to 4 mL/min/1.73 m^2^, necessitating hemodialysis. Renal ultrasound findings, fractional excretion of sodium, and urinalysis suggested acute kidney injury. Renal biopsy showed acute tubular injury, tubular red blood cell casts, and an absence of active glomerulonephritis, similar to the pathological findings of warfarin related nephropathy. A diagnosis of anticoagulant related nephropathy secondary to dabigatran was therefore established. This case demonstrates that dabigatran, like warfarin, may increase tubular bleeding risk in patients, irrespective of underlying kidney or glomerular disease.

## 1. Introduction

Anticoagulant related nephropathy is a form of acute kidney injury caused by excessive anticoagulation [1–3]. While traditionally associated with warfarin [1, 4, 5], direct oral anticoagulants (DOACs) have been shown to be related to this type of kidney injury [3]. The diagnosis of anticoagulant related nephropathy is based on histopathological findings: acute tubular injury, tubular red blood cell casts visualized on light microscopy, and absence of acute glomerular disease [3]. Intrarenal bleeding in the setting of anticoagulation leads to tubular obstruction by red blood cells and subsequent kidney injury [1, 3]. Further cellular damage is also likely caused by heme toxicity [6].

Overall incidence of DOAC related nephropathy is unknown with the exception of a few case reports [7]. The direct thrombin inhibitor, dabigatran, is the most commonly reported DOAC associated with anticoagulant related nephropathy [8]. Apixaban, a Factor Xa inhibitor, has also been linked to anticoagulant related nephropathy [9]. All known cases of dabigatran related nephropathy have occurred in patients who also had underlying IgA nephropathy suggesting that glomerular disease, and IgA nephropathy in particular, is a risk factor for dabigatran related nephropathy [7]. Herein we describe a case of a biopsy-proven dabigatran related nephropathy in a patient with no prior history of IgA nephropathy, further supporting the notion that DOACs may induce kidney injury in the absence of significant underlying kidney disease in a manner similar to warfarin.

## 2. Case Presentation

An 81-year-old woman with multiple medical comorbidities, significant for atrial fibrillation and anticoagulated with dabigatran 150 mg twice a day, presented with acute on chronic kidney injury in the setting of volume overload. Dabigatran was started two years prior. Her medical profile also included coronary artery disease, stage 2A chronic kidney disease, insulin dependent diabetes mellitus, hypertension, asymptomatic chronic lymphocytic leukemia, hypothyroidism, and a stable pulmonary nodule. Her medications included aspirin 81 mg once a day, amlodipine 5 mg once a day, losartan 150 mg once a day, hydrochlorothiazide 37.5 mg once a day, bisoprolol 7.5 mg once a day, nitroglycerin patch 0.4 mg from 8 a.m. to 8 p.m. per day, metformin 500 mg four times a day, insulin glargine 70 units twice a day, insulin Humalog 30-60 units as per sliding scale three times a day with meals, and levothyroxine 125 microgram once a day. She did not have significant alcohol or smoking history.

The patient presented with a one-week history of progressive dyspnea. She denied cough, fevers, chills, or sick contacts. Review of systems was otherwise unremarkable. On presentation, she was hypoxemic, oxygen saturation ranging from 88% to 92% on 6L of oxygen via nasal prongs. She was hemodynamically stable, afebrile and the remainder of her vital signs was within normal limits. On examination, her jugular venous pressure was measured at 6 cm above the sternal angle. There were no murmurs, extra heart sounds, heaves, or thrills. She had bilateral pitting edema of her legs extending to the knees. Auscultation of the lungs revealed decreased breath sounds bilaterally with coarse crackles. The remainder of her physical examination was otherwise unremarkable.

Initial labs showed leukocytosis of 16.8 (reference range: 4-10 X 10^9^/L) (see Table 2) with a lymphocyte count of 11.3 (reference range: 0.5-3.3 X 10^9^/L), neutrophil count of 5.4 (reference range: 2.0 – 9.0 X 10^9^/L), and monocyte count of 0.2 (reference range: 0.0 – 1.0 X 10^9^/L). Hemoglobin was 118 g/L (reference range: 123-157 g/L); platelets were 217 (reference range: 130-400 X 10^9^/L) (see Table 2). Her BNP was elevated at 5741 (reference range for age > 75: 300 – 1800 ng/L) (see Table 2). Serum creatinine was 177 umol/L (reference range: 40-100 umol/L) with an eGFR of 23 mL/min/1.73 m^2^ (reference range: ≥ 60 mL/min/1.73 m^2^). At baseline, serum creatinine was 91 umol/L with eGFR 57 mL/min/1.73 m^2^. Electrolytes were within normal limits (see Table 2). INR was elevated at 1.6 (reference range: 0.8 – 1.1) with an elevated activated Partial Thromboplastin Time (aPTT) of 50 seconds (reference range: 28-38 seconds) (see Table 2).

Chest x-ray showed interstitial pulmonary edema with stable cardiomediastinal contours. There were trace pleural effusions. Transthoracic echocardiogram showed normal biventricular size and systolic function. Ejection fraction was > 60%. There were mild left ventricular hypertrophy and no valvular disease.

**Figure 1:**
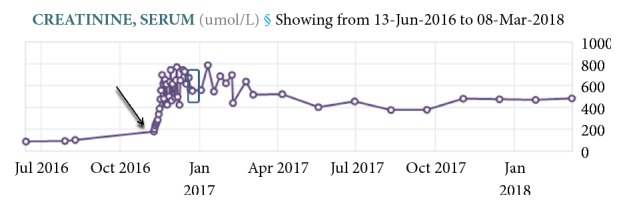
***Serum creatinine trend.*** Serum creatinine at baseline was 91 umol/L (reference range: 40-100 umol/L). It increased to 177 umol/L on day of admission (arrow) to peak of 800 umol/L during admission. On day of discharge, serum creatinine was 554 umol/L (box). Dabigatran was started two years before this episode.

The patient was diuresed with furosemide 40 mg IV twice a day for a total dose of 80 mg IV within the first day and placed on fluid restriction of <2 L/day. Her dyspnea and hypoxemia resolved within 24 hours. However, her serum creatinine continued to increase. Her serum creatinine rose from a baseline of 91 umol/L (reference value: 40-100 umol/L) to 177 umol/L on day of admission and reached 618 umol/L within 7 days of hospitalization (Figure 1). Furosemide was discontinued after the first day of admission. With the rise of creatinine, fluid restriction was discontinued and instead, isotonic intravenous fluids were administered, with no change in serum creatinine. She was referred to the nephrology service for assessment of acute kidney injury. Renal replacement therapy was initiated via a central venous catheter and intermittent hemodialysis. Dabigatran was held.

Urinalysis demonstrated cloudy and brown-colored urine, but no gross hematuria by inspection. Microscopic analysis showed >30 dysmorphic red blood cells/high powered field, protein > 5 g/L, and white blood cells (WBC) > 5/high powered field, while leukocyte esterase and nitrites were negative (see Table 1). Before this hospitalization, there was no evidence of hematuria or proteinuria on urinalysis. Renal ultrasound showed bilateral, mild increase in renal parenchymal echogenicity, suggesting medical renal disease. There was no hydronephrosis. Fractional excretion of sodium (FeNa) was 1.1%. Anti-Streptolysin O titer was 449 IU/mL (reference values: 0-200 IU/mL) and serum IgA was 5.85 g/L (reference values: 0.60-4.20 g/L) (see Table 2). Complement levels, C3 and C4, were normal (see Table 2). Antinuclear antibodies, anti-double stranded DNA antibodies, anti-cyclic citrullinated peptide antibodies, anti-glomerular basement membrane antibodies, cytoplasmic anti-neutrophil cytoplasmic antibodies (c-ANCA), and perinuclear anti-neutrophil cytoplasmic antibodies (p-ANCA) were normal (see Table 2). Serum free light chains ratio (kappa: lambda) was also mildly elevated at 3.16 (reference values: 0.26-1.65) (see Table 2). Albumin to creatinine ratio was 15.37 mg/mmol (reference value <2.29 mg/mmol). HIV and hepatitis serology were negative.

**Figure 2:**
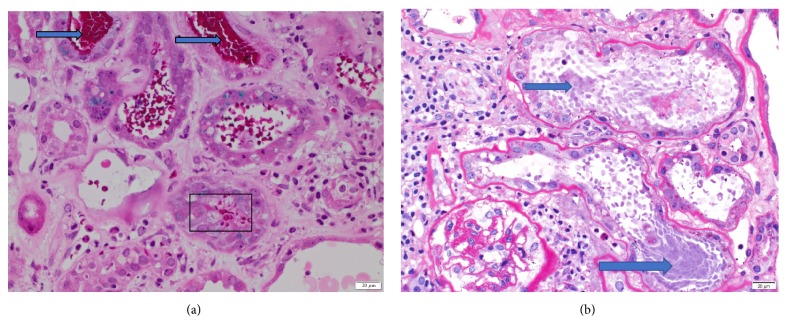
***Kidney biopsy histopathology I.*** (a) Kidney biopsy shows presence of abundant RBCs (box) and RBC casts (arrows) (Periodic Acid Schiff stain, Light Microscopy, Magnification: X200). (b) Kidney biopsy shows acute tubular injury with loss of brush borders, attenuated tubular epithelium, and reactive epithelial changes. RBC casts (arrows) indicate significant hemorrhage in tubules. The mesangium of glomerulus is minimally expanded with no evidence of proliferation. (Periodic Acid Schiff Stain, Light Microscopy, Magnification: X200.)

**Figure 3:**
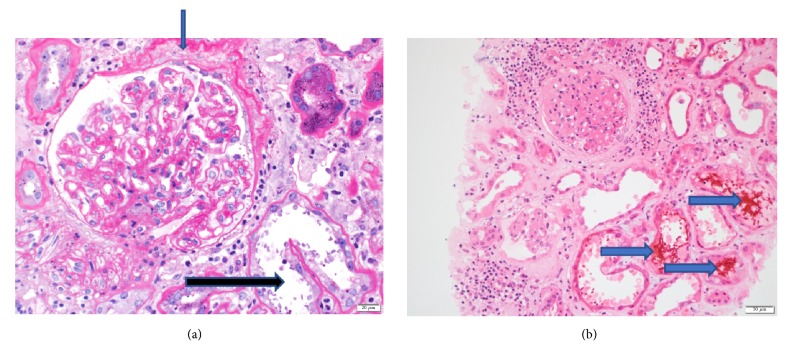
***Kidney biopsy histopathology II.*** (a) Periodic Acid Schiff (Magnification: X200) staining of glomeruli shows minimal mesangial expansion with no evidence of mesangial and endocapillary proliferation. Chronic ischemic damage is also evident by thickening of Bowman capsule membrane (blue arrow). The damaged tubules (black arrow) are also shown. (Light Microscopy.) (b) Hematoxylin and Eosin staining (Magnification: X100) shows damaged tubules with RBC casts (arrows) being seen. There is an absence of active glomerulonephritis.

**Figure 4:**
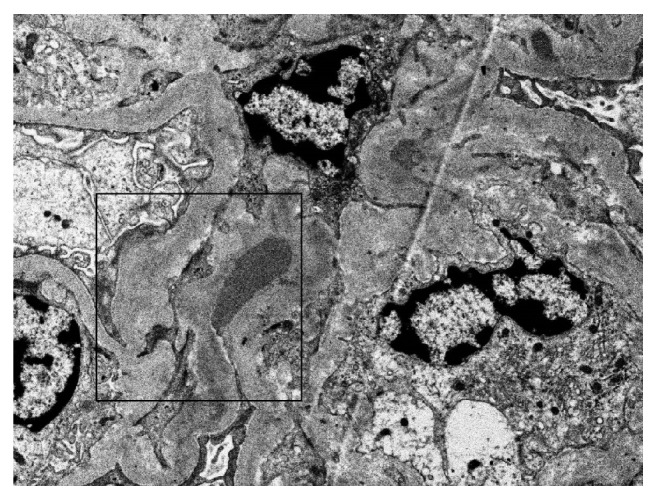
***Kidney Biopsy Electron microscopy.*** Small subepithelial electron dense deposit shown in the mesangial notch (box) (Electron Microscopy).

**Figure 5:**
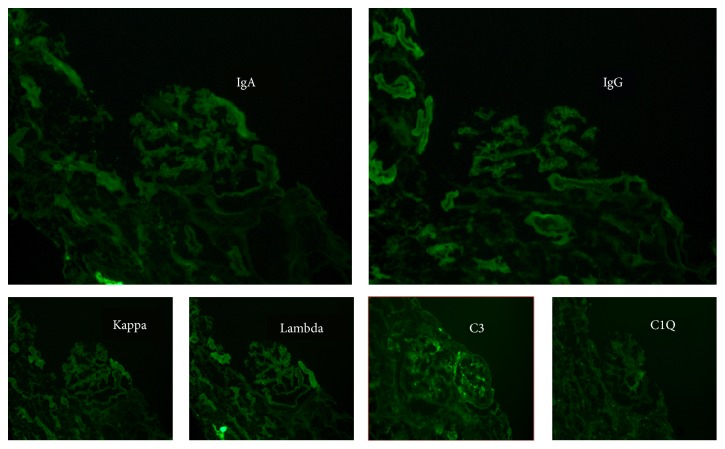
***Kidney biopsy immunofluorescence*.** Immunofluorescence microscopy probing for IgA, IgG, Kappa light chains, Lambda light chains, C3, and C1Q deposits. Trace focal deposition of C3 is present. IgA, IgG, Kappa light chains, Lambda light chains, and C1Q deposits are not visualized.

The patient underwent a diagnostic kidney biopsy, which showed extensive red blood cell casts within tubular lumen as well as tubular epithelial cell injury on light microscopy (Figures 2(a) and 2(b)), without evidence of acute glomerulonephritis (Figures 3(a) and 3(b)). Electron microscopy showed subepithelial “hump-like” deposits most of which were located within the mesangial notch areas, and thus, thought to be resolving postinfectious glomerulonephritis (Figure 4). Immunofluorescence microscopy was negative for IgA and IgG, but trace positive for C3 (Figure 5). The findings were most consistent with anticoagulant-related nephropathy with concurrent resolving postinfectious glomerulonephritis.

The patient had a protracted stay in hospital. During her admission, she was found to have enterococcus faecium urinary tract infection which responded to vancomycin and subsegmental pulmonary emboli treated with unfractionated heparin. She did not have recovery of her renal function and continued intermittent hemodialysis.

## 3. Discussion

Anticoagulant related nephropathy has characteristic histopathological findings: intratubular hemorrhage, tubular red blood cell (RBC) casts, and tubular epithelial cell injury seen on light microscopy which exist in the absence of active glomerulonephritis [2, 3]. The histological findings of our patient matched these typical lesions, establishing a diagnosis of anticoagulant related nephropathy secondary to dabigatran.

The kidney biopsy showed acute tubular injury associated with intratubular RBC and RBC casts, on the background of mild-moderate chronic tubulointerstitial disease (Figures 2(a), 2(b), 3(a), and 3(b)). There were focal subepithelial electron dense deposits found in the mesangial notch on electron microscopy (Figure 4) suggesting resolved postinfectious glomerulonephritis; however no active glomerulonephritis or IgA nephropathy was present. Thus, the acute deterioration in kidney function could not be attributed to glomerulonephritis, but rather injury related to intratubular bleeding. The finding of a few dense deposits consistent with resolving postinfectious glomerulonephritis was likely a bystander; though it is possible that this lesion predisposed the patient to glomerular and subsequent intratubular bleeding. Preadmission urinalysis showed no evidence of hematuria or proteinuria.

Both warfarin and dabigatran related nephropathies appear identical on biopsy [10]. Excessive anticoagulation from both medications leads to severe tubular hemorrhage, which in turn causes tubular obstruction by red blood cell casts [11]. Subsequently, there is tubular epithelial injury as well as heme toxicity and iron-associated cellular damage [6, 12, 13]. Warfarin inflicts direct glomerular damage by inhibiting vitamin k-dependent proteins, matrix G1a protein, and the growth arrest specific genes 6 (GAS-6) [14]. GAS-6 is involved in inhibition of vascular calcification and vascular smooth muscle cell migration and apoptosis [15]. With its inhibitory effect blocked by warfarin, there is progression of smooth muscle cell apoptosis and calcification [11]. Dabigatran reduces thrombin activity which in turn can affect protease activated receptor 1 (PAR-1) activity [8, 11]. PAR-1 is a G-protein coupled thrombin receptor expressed on endothelial cells that participate in endothelial function, vascular permeability, leukocyte migration, and adhesion [10, 16, 17]. Reduced PAR-1 activity can alter vascular endothelial tone, disrupt the endothelial monolayer integrity, and alter the glomerular filtration barrier [16, 17]. Thus, both warfarin and dabigatran likely share a common pathway of kidney injury [8, 11]: disruption of the glomerular filtration barrier, intratubular bleeding, and tubular obstruction by red blood cells, heme free radical-related cytotoxicity, and ultimately tubular epithelial cell injury [5, 8, 11].

The current data on dabigatran related nephropathy suggests that IgA nephropathy may be a predisposing factor [7]. This is based on few case reports, all of which featured patients with underlying diagnosis of IgA nephropathy [7]. This was not the case for our patient. Instead, our patient's presentation resembled the clinical picture of warfarin related nephropathy [4]. Warfarin related nephropathy has been seen in patients with and without chronic kidney disease; though the latter has been found to be a significant risk factor [18, 19]. Other risk factors are advanced age, hypertension, diabetes mellitus, diabetic nephropathy [18], heart failure, and coronary vascular disease [18, 20]. Medications associated with increased risk are aspirin, calcium channel blockers, and ACE inhibitors [11, 18]. Thus, based on the warfarin literature [8, 21], underlying kidney disease, in this case, stage 2a chronic kidney disease and resolving postinfectious glomerulonephritis, may have predisposed our patient to acquire dabigatran related nephropathy. Moreover, our patient's comorbidities, hypertension, heart failure, diabetes mellitus, and coronary artery disease, medications including aspirin and amlodipine, in the setting of advanced age and long-term dabigatran usage may have contributed in a potentially additive manner to the susceptibility for anticoagulant related nephropathy.

There is limited data on dabigatran related nephropathy compared to warfarin, which has been widely studied since its first identification in 2009 [1]. Warfarin related nephropathy can accelerate the progression of chronic kidney disease [18, 22]. It is also correlated with increased mortality [20]. Furthermore, rat models have shown that the stage of chronic kidney disease is directly related with the risk of acquiring warfarin related nephropathy [23]. Similar data regarding mortality implications and kidney disease progression are not yet available for dabigatran related nephropathy [7]. Further research is required to understand the incidence of biopsy proven dabigatran related nephropathy; however, it is likely to be rare given the widespread and increasing use of DOAC in clinical care. Nevertheless, increasing case reports are emerging including recently with the factor Xa inhibitor, apixaban [9]. As the use of DOACs increases, it will be important to define not only the incidence of acute kidney injury, but also the potential risk factors, whether independent or additive, as well as mortality and morbidity implications.

## 4. Conclusion

Our case illustrates the novel finding that dabigatran related nephropathy can occur without underlying IgA nephropathy or significant prior renal disease, similar to warfarin related nephropathy [3]. This widens the differential diagnosis for acute renal failure; especially, when the index of suspicion is high for renal etiology in the setting of DOAC usage. Early detection of this diagnosis and timely cessation of the offending medication has the potential to prevent significant kidney disease progression and the need for renal replacement therapy. We postulate that the presence of comorbidities may serve as additive risk factors. Future directions for research include studies to quantify the incidence and prevalence of this condition and stratify risk factors, and outcomes related to this drug complication.

## Figures and Tables

**Table 1:** Urine analysis.

Test	Result	Ref. Range	Abnormality
**Color**	**Brown**		Abnormal
**Appearance**	**Cloudy**		Abnormal
**Specific Gravity, Urine**	1.015	<=1.030	
**pH**	6.0	5.0-8.5	
**Leukocyte**	Negative	Negative	
**Nitrite**	Negative	Negative	
**Protein**	**>=5.0**	Negative (g/L)	Abnormal
**Glucose**	**8.3**	Negative	Abnormal
**Ketones**	Negative	Negative	
**Blood**	**Large**	Negative	Abnormal
**WBC**	**Present**	0-5 (/HPF)	Abnormal
**Urine RBC**	**>30**	0-5 (/HPF)	Abnormal
**Epithelial Cells**	Present	(/HPF)	
**Amorphous Material**	Present	(/HPF)	
**Urine Bacteria**	**Present**	(/HPF)	Abnormal
**Hyaline Casts**	Present	(/LPF)	

**Table 2:** Lab investigations.

Test	Result	Ref. Range	Abnormality
**Serum Sodium**	137 mmol/L	133 – 145 mmol/L	
**Serum Potassium**	4.5 mmol/L	3.3 – 5.1 mmol/L	
**Serum Chloride**	104 mmol/L	98 – 111 mmol/L	
**Serum Bicarbonate**	19 mmol/L	21 – 31 mmol/L	
**Serum Creatinine**	**177 umol/L**	35 – 100 umol/L	Abnormal
**Hemoglobin**	118 g/L	123 – 157 g/L	
**White Blood Count**	**16.8 X 10** ^**9**^ **/L**	4 – 10 X 10^9^/L	Abnormal
**Platelet**	217 X 10^9^/L	130 – 400 X 10^9^/L	
**INR**	**1.6**	≤ 1.1	Abnormal
**aPTT **	**50 seconds **	28 – 38 seconds	Abnormal
**Antinuclear Antibodies, Anti DS- DNA antibodies, **	Negative		
**C-ANCA, P-ANCA**	Negative		
**Anti-Glomerular Basement Membrane Antibody**	Negative		
**C3, C4**	Normal		
**HIV, Hepatitis serology**	Negative		
**Serum IgA**	**5.85 g/L **	0.60 – 4.20 g/L	Abnormal
**Anti-Streptolysin O titer**	**449 IU/mL**	0-200 IU/mL	Abnormal
**Free Kappa**	**223 mg/L**	3.3 – 19.4 mg/L	Abnormal
**Free Lambda**	**70.50 mg/L**	5.71 – 26.3 mg/L	Abnormal
**Kappa: Lambda Ratio**	**3.16**	0.26 – 1.65	Abnormal
